# Analysis of Natural Variation in Bermudagrass (*Cynodon dactylon*) Reveals Physiological Responses Underlying Drought Tolerance

**DOI:** 10.1371/journal.pone.0053422

**Published:** 2012-12-28

**Authors:** Haitao Shi, Yanping Wang, Zhangmin Cheng, Tiantian Ye, Zhulong Chan

**Affiliations:** 1 Key Laboratory of Plant Germplasm Enhancement and Specialty Agriculture, Wuhan Botanical Garden, Chinese Academy of Sciences, Wuhan, China; 2 University of Chinese Academy of Sciences, Beijing, China; University of Nottingham, United Kingdom

## Abstract

Bermudagrass (*Cynodon dactylon*) is a widely used warm-season turfgrass and one of the most drought tolerant species. Dissecting the natural variation in drought tolerance and physiological responses will bring us powerful basis and novel insight for plant breeding. In the present study, we evaluated the natural variation of drought tolerance among nine bermudagrass varieties by measuring physiological responses after drought stress treatment through withholding water. Three groups differing in drought tolerance were identified, including two tolerant, five moderately tolerant and two susceptible varieties. Under drought stress condition, drought sensitive variety (Yukon) showed relative higher water loss, more severe cell membrane damage (EL), and more accumulation of hydrogen peroxide (H_2_O_2_) and malondialdehyde (MDA), while drought tolerant variety (Tifgreen) exhibited significantly higher antioxidant enzymes activities. Further results indicated that drought induced cell injury in different varieties (Yukon, SR9554 and Tifgreen) exhibited liner correlation with leaf water content (LWC), H_2_O_2_ content, MDA content and antioxidant enzyme activities. Additionally, Tifgreen plants had significantly higher levels of osmolytes (proline level and soluble sugars) when compared with Yukon and SR9554 under drought stress condition. Taken together, our results indicated that natural variation of drought stress tolerance in bermudagrass varieties might be largely related to the induced changes of water status, osmolyte accumulation and antioxidant defense system.

## Introduction

Drought is one of the most serious world-wide problems and largely affects plant growth, development and survival rate, leading to enormous crop yield loss. Plants are greatly restricted in growth field under stress condition, and have evolved many mechanisms to rapidly adapt to drought stress condition to keep growth and productivity [1], [2]. Recently, more attentions have been paid to mechanisms of plant drought stress tolerance, including physiological and biochemical metabolisms, gene expression regulation, proteomic profiling and cross-talks between several hormones etc, which further helps us develop different genetic approaches to improve plant drought tolerance and prevent yield loss.

In response to drought stress, turfgrass has developed complex mechanisms such as physiological, biochemical, molecular and cellular changes to cope with limited water supply [3], [4]. Comparatively, bermudagrass (*Cynodon dactylon*) is one of the most drought tolerant turfgrasses [5], [6]. As a warm-season perennial grass, bermudagrass is widely used as turfgrass on sport fields, golf courses and home lawns. Drought stress is a world-wide problem, and different grass species may develop different strategies to tolerate, escape, or avoid drought stress condition [4], [7], [8], [9], [10].

To date, a lot of research groups have paid attentions to the natural variations in biotic and abiotic stress tolerances in many plant species, such as *Arabidopsis*, rice, bermudagrass, *brachypodium ditachyon*, and perennial ryegrass (*Lolium perenne*) [3], [9], [11], [12], [13], [14], [15]. Using these germplasms differing in drought tolerance, the major quantitative trait locis (QTL) contributing to drought tolerance and genetic networks controlling important stress processes were further dissected [4], [5], [9], [10], [15]. Previous studies have suggested that drought tolerance of bermudagrass varieties might be correlated with plant development such as leaf firing, root and shoot systems, mass production [16], [17], [18], [19], accumulation of dehydrin [9], [20], evapotranspiration [21], leaf water content (LWC), chlorophyll content, proline content, and antioxidant enzyme activities [10], [22], [23]. Physiological and biochemical mechanisms in response to water deficit stress have been largely studied in some bermudagrass varieties, however, most of these studies have focused on physiological level. Correlations between the natural variation and the detailed mechanisms among different species are still largely unknown.

The objectives of this study were to observe the genetic variations of several bermudagrass varieties in response to drought stress and to investigate possible mechanisms involved in drought stress tolerance variation. Nine bermudagrass varieties were collected and the natural variations of drought tolerance were evaluated. The relationships betweendrought tolerances and several physiological parameters were further comparatively dissected and discussed. These results provided some insights to understand the genetic and molecular mechanisms of bermudagrass drought tolerance.

## Results

### Drought tolerance evaluation of nine bermudagrass varieties

To compare the drought tolerance of nine bermudagrass varieties, healthy plants were subjected to well-watered condition and drought condition (soil water deficit) by withholding water in the soil for 28 d. After drought stress treatment, the LWC, leaf electrolyte leakage (EL) and the survival rate were examined to evaluate the drought tolerance. Under control condition, the LWC, EL and survival rate among all varieties were maintained almost at the same level (Table 1). When the drought stress was applied for 28 d, the LWC and survival rates of all varieties were largely decreased, while the ELs were increased. However, the changes of these parameters varied with varieties (Table 1). Tifgreen and Tifway had the highest LWC and survival rate but the lowest EL under drought stress condition, while Yukon and Wranger had the lowest LWC and survival rate but the highest EL. All these parameters (LWC, EL and survival rate) of other five varieties (LaPaloma, Riviera, SR9554, Laprima and Veracruz) were between the above four varieties (Table 1).

**Table 1:** Experiment ID, accession, drought tolerant (DT), Leaf water content (LWC), electrolyte leakage (EL) and survival rate of nine bermudagrass varieties.

			LWC (%)		EL (%)		Survival rate (%)	
ID	Accession	DT	Control	Drought	Control	Drought	Control	Drought
8	Tifgreen	T	78.37±0.34	54.27±2.25	17.26±0.93	49.12±1.37	100	58.23±2.44
9	Tifway	T	78.19±0.53	50.64±2.17	14.71±0.69	53.83±1.87	100	60.79±3.43
5	LaPaloma	M	78.94±0.48	36.61±1.38	14.43±0.93	64.17±2.84	100	15.56±6.30
7	Riviera	M	78.44±0.51	37.31±1.01	16.01±1.45	68.58±1.98	100	12.38±3.66
2	SR9554	M	79.55±0.69	30.28±1.93	14.03±1.63	64.99±2.78	100	9.60±2.47
4	Laprima	M	79.62±0.67	31.87±1.36	15.81±0.78	67.68±2.25	100	13.65±4.38
6	Veracruz	M	79.23±0.49	27.65±1.23	16.53±1.01	67.98±3.69	100	6.35±4.10
1	Wrangler	S	79.31±0.51	17.27±1.77	18.21±1.48	76.66±2.30	100	0
3	Yukon	S	78.80±0.39	15.93±1.85	16.53±1.63	86.74±2.08	100	2.02±1.02

T, tolerant; M, moderately tolerant; S, Susceptible. Data are means ± SE (n≥7).

**Figure 1:**
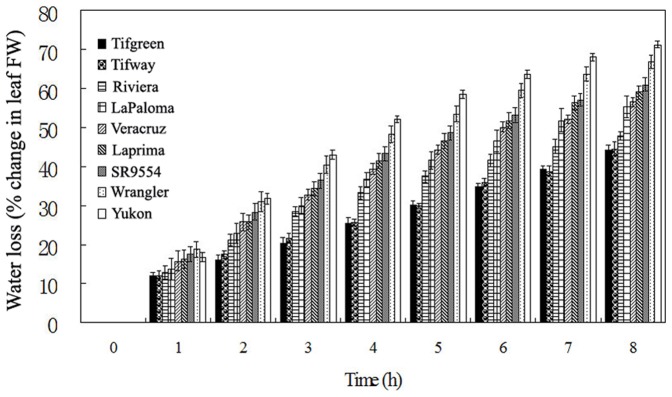
Analysis of leaf water loss (% change in leaf FW) in nine bermudagrass varieties. The results shown are mean ± SE (n = 8).

To further confirm the above results, we also detected the leaf water loss (% change in leaf fresh weight (FW)) of different bermudagrass varieties. As shown in Figure 1, with the detach time increasing from 0 h to 8 h, Tifway and Tifgreen showed the least water loss, while Yukon and Wranger exhibited the most. Other five varieties were in between the above four varieties. These results were consistent with those parameters of the stress tolerance test (Table 1).

### Groups of bermudagrass varieties differing in sensitivity to drought stress

**Figure 2:**
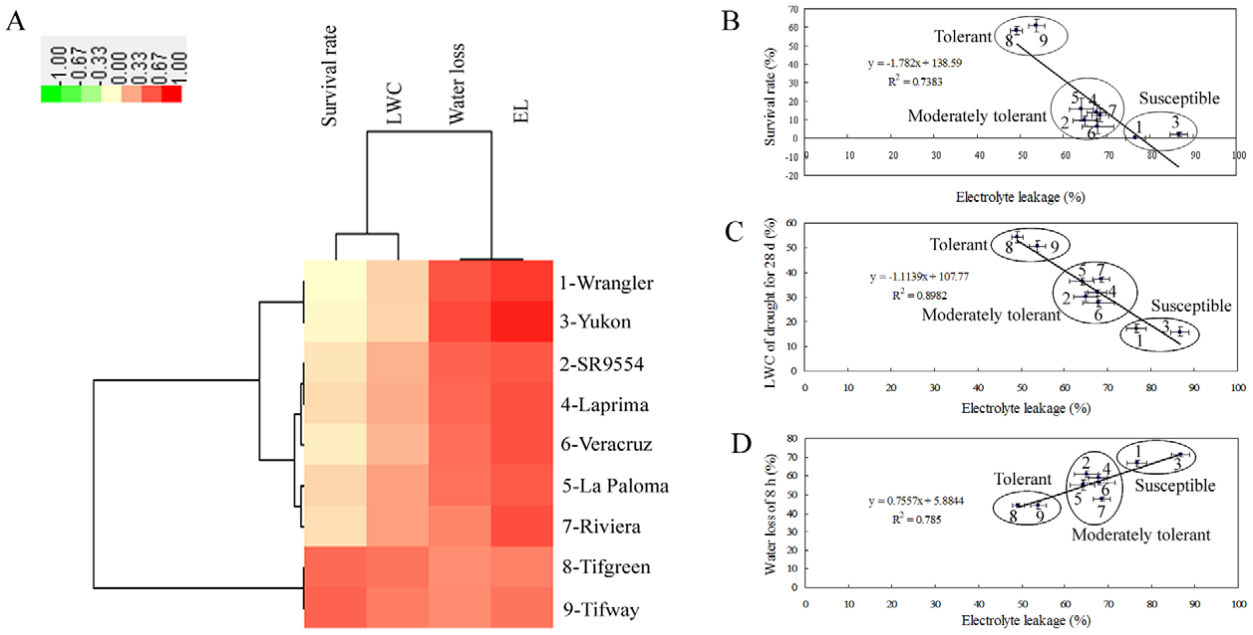
Groups of bermudagrass varieties differing in sensitivity to drought stress. (A) Hierarchical cluster analysis of the groups of bermudagrass varieties differing in sensitivities to drought stress based on the data of EL, survival rate, LWC after drought treatment for 28 d and water loss at 8 h after detachment. Resulting tree figure was displayed using the software package and Java Treeview. (B)–(D) Nine bermudagrass varieties were clustered to three groups differing in sensitivity to drought stress based on the data of EL and survival rate after drought treatment for 28 d (B), EL and LWC after drought treatment for 28 d (C), and EL at 28 d after drought and water loss at 8 h after detachment (D). The numbers of 1, 2, 3, 4, 5, 6, 7, 8 and 9 indicate the variety of Wranger, SR9554, Yukon, Laprima, LaPaloma, Veracruz, Riviera, Tifgreen and Tifway, respectively. The results shown are mean ± SE (n≥7).

Among nine bermudagrass varieties used in this study, when subjected to drought stress for 28 d, LWC ranged from 15.93% to 54.21%, EL ranged from 49.12% to 86.74%, and survival rate ranged from 0 to 60.79%, respectively (Table 1). Additionally, the leaf water loss of these nine varieties at 8 h after detachment ranged from 44.25% to 71.25% (Figure 1). Half-life of water loss was 3–4 h for drought sensitive varieties (Yukon and Wranger), and longer than 8 h for drought tolerant ones (Tifway and Tifgreen) after detachment (Figure 1). Based on the variations of above mentioned physiological parameters (Table 1 and Figure 1), several groups differing in their sensitivities to drought stress were identified through hierarchical cluster analysis (Figure 2A–D). Interestingly, although different parameters were chosen, all nine varieties were clustered into three same groups, namely tolerant, moderately tolerant and susceptible (Figure 2A–D). Two varieties (Tifgreen and Tifway) were the most tolerant ones, with least reduction in LWC and leaf water loss *in vitro*, lowest EL but highest survival rate under drought stress condition. Five varieties (LaPaloma, Riviera, SR9554, Laprima and Veracruz) were clustered in the moderately drought tolerant group, with moderate level changes of the above mentioned physiological parameters. The other two varieties (Yukon and Wranger) were reasonably identified as the susceptible materials. These results suggested a linkage between plant physiological changes and drought tolerance in bermudagrass.

### Comparison of water status and electrolyte leakage of different bermudagrass varieties under drought stress condition

**Figure 3:**
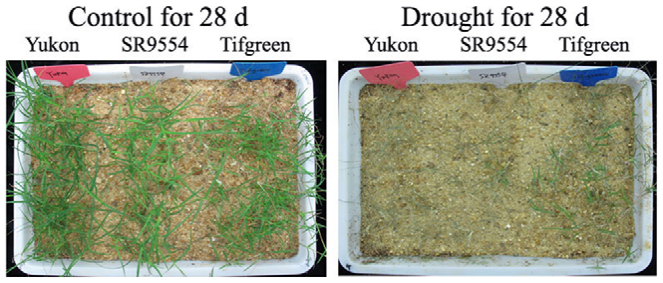
Plant responses to drought stress. Three varieties representing drought tolerant, moderately tolerant and susceptible groups that were compared under drought stress condition. The plants with nearly the same crown size were subjected to control watering condition and drought condition for 28 d.

To further investigate the possible mechanisms of the drought tolerance variations, three bermudagrass varieties (Tifgreen, SR9554 and Yukon) differing in sensitivities to drought stress were chosen for the following experiments. Leaf firing, also known as the change of leaf chlorosis, is a good assessment of turfgrass drought tolerance in soil [21], [23]. As described previously [21], [23], leaf firing was visually accessed by counting the grass leaves that turned yellow and brown. Under well-watered conditions, all of Tifgreen, SR9554 and Yukon showed very little leaf firing. After withholding water for 28 d, Yukon showed the most severe phenomena of leaf firing, while the leaf tissues of Tifgreen were much greener when compared with the other two varieties (Figure 3).

**Figure 4:**
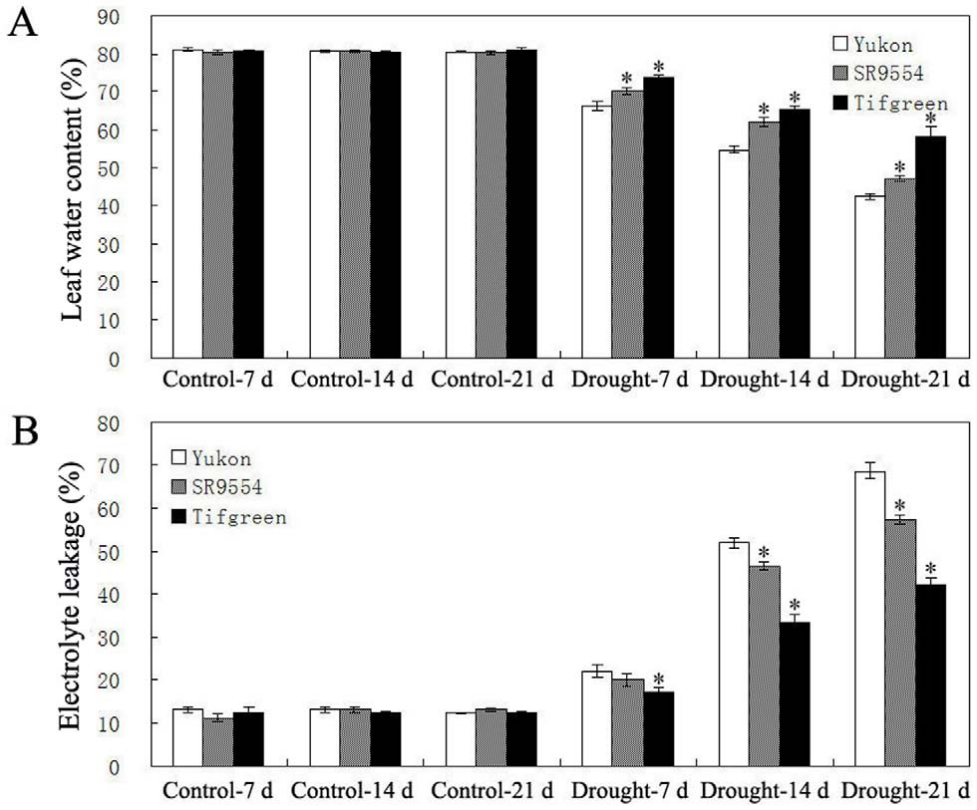
Water status and cell membrane damage of three bermudagrass varieties differing in drought tolerance. LWC (A) and EL (B) of Tifgreen, SR9554 and Yukon varieties during drought stress were shown here. The results shown are mean ± SE (n = 8). Asterisk symbols indicate significant differences from Yukon (*P*<0.05).

Under control condition, all three bermudagrass varieties (Tifgreen, SR9554 and Yukon) maintained the LWC at about 80% (Figure 4A). After drought stress treatment, the LWC exhibited a gradual decline in all three varieties (Figure 4A). However, LWC of Yukon decreased from 66% to 42% from 7 d to 21 d after drought stress treatment, while from 69% to 47% for SR9554, and from 74% to 58% for Tifgreen, respectively, demonstrating that Tifgreen showed the slowest water loss under drought stress condition (Figure 4A).

Consistent with the change of LWC, all plants showed a gradual increase for EL after drought stress treatment. However the extent of change varied among different varieties (Figure 4B). In comparison with the others, Tifgreen exhibited significant lowest EL, while Yukon showed the highest EL after drought stress treatment from 7 d to 21 d (Figure 4B).

### Changes of proline and sugar contents under drought stress condition

**Figure 5:**
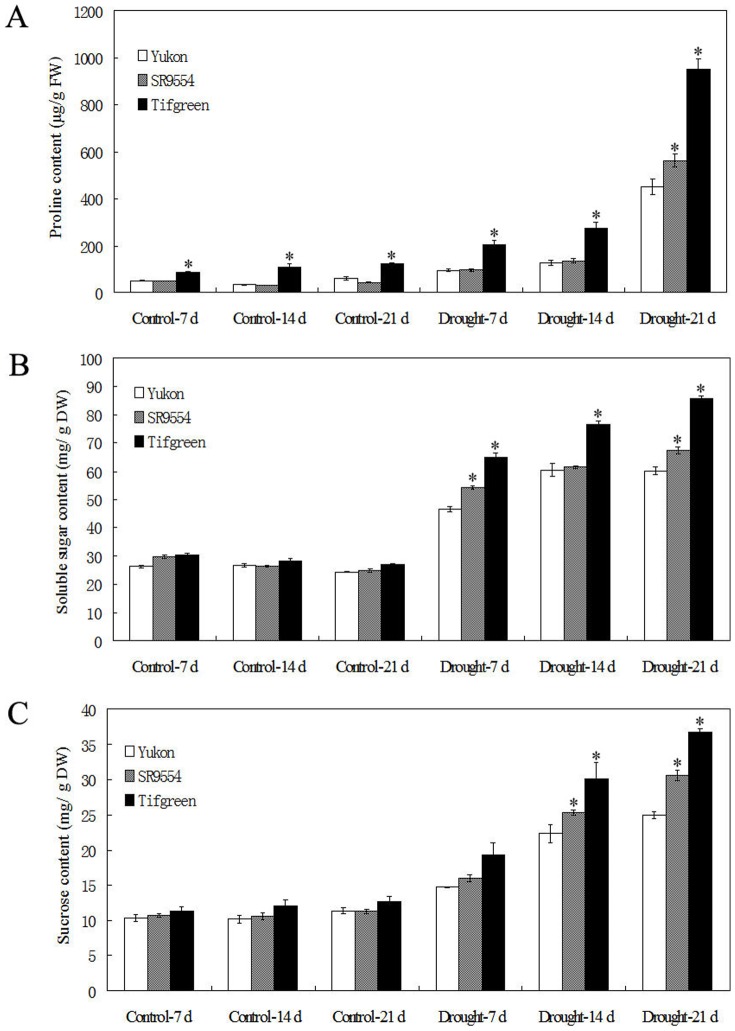
Accumulation of osmolytes of three bermudagrass varieties differing in drought tolerance during drought stress. Changes of proline content (A), soluble sugars (B) and sucrose content (C) of three genotypes of bermudagrass during drought stress were shown here. The results shown are mean ± SE (n = 4). Asterisk symbols indicate significant differences from Yukon (*P*<0.05).

Under control condition, the internal proline level of plant was very low, but was largely induced when subjected to drought stress treatment (Figure 5A). Interestingly, Tifgreen plant had significantly higher proline content when compared with those of Yukon and SR9554 varieties under well-watered condition (Figure 5A). After drought stress treatment, proline levels were obviously up-regulated in all three varieties. However, proline level of Tifgreen was significantly higher than those of the other two varieties from 7 d to 21 d after drought treatment. SR9554 also exhibited significantly higher level of proline than Yukon after drought treatment for 21 d (Figure 5A). In addition, the contents of soluble sugars were also largely induced in all three varieties after drought treatment (Figure 5B and C). Under control condition, no significant differences of sucrose and soluble total sugar levels were observed among three varieties (Figure 5B and C). When drought stress was applied, SR9554 had significantly higher levels of both sucrose and soluble total sugars, while Tifgreen accumulated the highest content of sugars (Figure 5B and C). Together, the changes of proline content and sugars might be partially contributed to enhanced drought tolerance of Tifgreen variety.

### Changes of ROS level and antioxidant enzyme activities in response to drought stress

**Figure 6:**
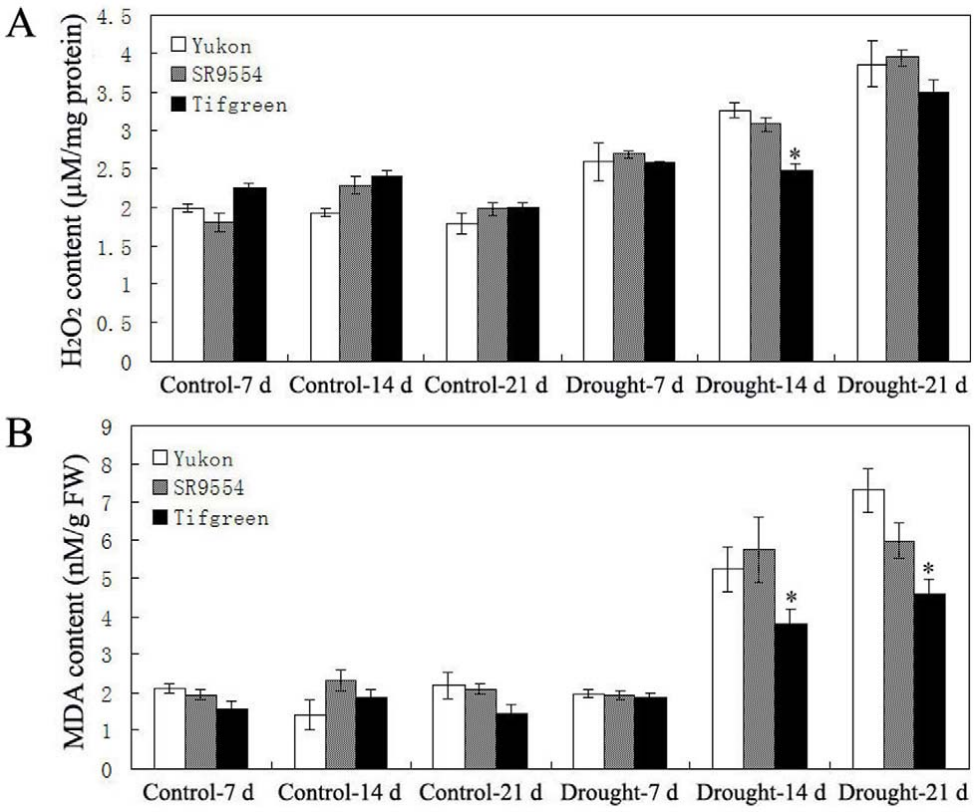
ROS level of three bermudagrass varieties differing in drought tolerance during drought stress. Changes of H_2_O_2_ content (A) and MDA level (B) of three varieties (Tifgreen, SR9554 and Yukon) differing in drought tolerance during drought stress were shown here. The results shown are mean ± SE (n = 4). Asterisk symbols indicate significant differences between Yukon and other genotypes (*P*<0.05).

As two major indicators for ROS level and oxidative damage, hydrogen peroxide (H_2_O_2_) and malondialdehyde (MDA) contents were assayed in this study [15]. As shown in Figure 6, all three varieties showed nearly the same levels of H_2_O_2_ and MDA under well-watered condition from 7 d to 21 d (Figure 6A and B). After drought treatment, gradual increases of H_2_O_2_ and MDA contents were observed in all varieties (Figure 6A and B). After 14 d and 21 d of drought stress treatments, the H_2_O_2_ and MDA contents in Tifgreen were significantly lower than those in Yukon (Figure 6A and B).

**Figure 7:**
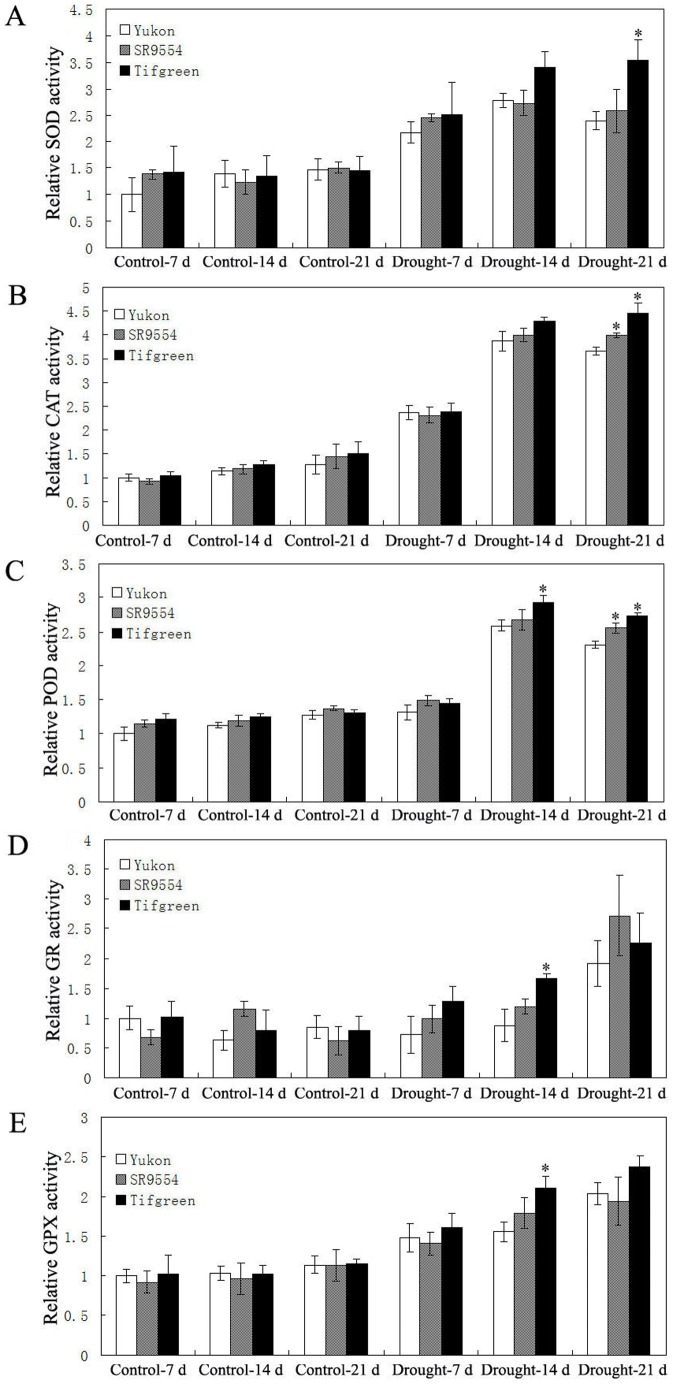
Antioxidant enzyme activities of three bermudagrass varieties differing in drought tolerance during drought stress. (A)–(E) Comparisons of SOD (A), CAT (B), POD (C), GR (D) and GPX (E) activities three represent drought tolerant, moderately tolerant and susceptible accessions under control condition and drought stress were shown here. The relative activities were quantified as fold change in comparison with Yukon under control conditions for 7 d. The results shown are mean ± SE (n = 4). Asterisk symbols indicate significant differences between Yukon and other genotypes (*P*<0.05).

To address the relationship between the changes of ROS level and the antioxidant enzymes activities, five antioxidant enzymes (superoxide dismutase (SOD), catalase (CAT), peroxidase (POD), glutathione reductase (GR), glutathione peroxidase (GPX)) activities were analyzed. Under control condition, the activities of five antioxidant enzymes (SOD, CAT, POD, GR and GPX) in all three varieties (Yukon, SR9554 and Tifgreen) showed no significant differences (Figure 7A–E). After drought stress treatment, Tifgreen exhibited significantly higher activities of five antioxidant enzymes than those of Yukon and SR9554 at some timepoints (Figure 7A–E).

### Correlation analyses of electrolyte leakage with other parameters under drought stress condition

**Figure 8:**
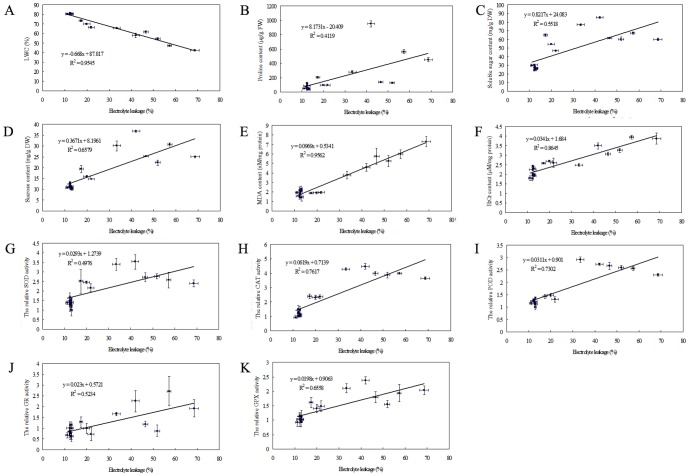
Correlations analysis of EL with other parameters during drought stress. (A)–(I) The linear correlation of EL with LWC (A), MDA content (B), Proline content (C), soluble sugars (D), sucrose content (E), H2O2 content (F), SOD activity (G), CAT activity (H), POD activity (I), GR activity (J) and GPX activity (K) of three represent cultivars (Yukon, SR9554 and Tifgreen) during drought stress. The results shown are mean ± SE (n≥4).

To elucidate the possible mechanisms of bermudagrass in response to drought stress, we analyzed the correlations of EL with other physiological parameters of three tested varieties (Yukon, SR9554 and Tifgreen) under drought stress condition. The LWC showed negative linear correlation with EL under drought stress condition (R^2^ = 0.95) (Figure 8A), suggesting that LWC was associated with drought induced cell injury in bermudagrass. However, there were no significant linear correlations between EL and the accumulations of osmolytes, including proline, soluble sugars and sucrose under drought stress condition (R^2^ = 0.42, 0.55, 0.66, respectively) (Figure 8B–D). Additionally, the MDA content, H_2_O_2_ content, CAT, POD and GPX activity of Yukon, SR9554 and Tifgreen were positively correlated with EL under drought stress condition (R^2^ = 0.96, 0.86, 0.76, 0.73 and 0.66, respectively) (Figure 8E–F, H–I, K). So the leaf water status, ROS level and activities of antioxidant enzymes might be associated with drought tolerance of bermudagrass.

## Discussion

Drought is one of the major abiotic stresses, which largely limits plant growth and yield, and induces various changes at cellular, physiological and metabolic levels. Selection of natural stress tolerant genotypes among different species is the lowest cost, the most efficient and rapidest method for plant breeding [3], [11], [12], [14]. To make full use of such strategy and develop new options, a comparative study of plant drought stress tolerances on the basis of physiological, biochemical and molecular changes is required [24]. Bermudagrass is a warm-season creeping grass and has natural variation in the drought response [5], [6]. Under drought stress condition, the upper parts of bermudagrass die off, but the grass will keep growing from its rhizomes [5], [6]. Additionally, bermudagrass grows and reproduces rapidly, and all these fundamental specifications make bermudagrass a potential target for genetic engineering of stress tolerant plants. In this study, we identified a wide range of variations of bermudagrass drastically differing in drought tolerance, and thoroughly detected some important parameters which were related with drought tolerance. Since turfgrasses consume large amounts of water around the world, these observations will give us some clues to breed drought-tolerant turfgrasses, as well as provide good opportunities to dissect drought stress response in other plant species. More importantly, because of its easy and quick reproduction, only litter survived bermudagrass (such as drought tolerant variety Tifgreen) during dry season can become a field of grasses during rainfall season.

Drought stress has direct impact on the disturbance of cell membrance [6], [9], [10]. EL, as one indicator of cell membrane stability, has been widely used to evaluate the extent of cell injury when subjected to various environment stresses [6], [9], [10], [15], [22], [23]. The LWC *in vivo* and leaf water loss *in vitro* implicate the water status of plant. The slower water loss during drought stress reflects the maintenance of plant water content [6], [9], [10], [15], [22], [23]. In this study, nine natural varieties of bermudagrass showed large variations in drought tolerance by evaluating cell membrane damage, water status and survival rate (Fig. 1; Table 1). Three groups of accessions differing in the drought tolerance were generally identified, with two tolerant varieties (Tifgreen and Tifway), five moderately tolerant varieties (LaPaloma, Riviera, SR9554, Laprima and Veracruz) and two susceptible varieties (Yukon and Wranger) (Fig. 2). Our results were consistent with Hu et al. [9] who found that Tifway was a drought tolerant bermudagrass material compared with the others. Besides, we also found that Tifgreen might have comparable drought tolerance with Tifway, and identified several new susceptible varieties (Yukon and Wranger) under drought condition. The difference of drought stress tolerance between Tifgreen and Yukon might be much more significant than previous identified bermudagrass varieties [9], [22], [23]. Therefore, the Yukon variety is also a very important material to reveal the mechanism of drought tolerance. The drastic natural variation can be further used to investigate the molecular, genetic, proteomic, metabolic basis of the drought response of bermudagrass. Further studies to identify drought-responsive candidate genes and examine transcriptomic and proteomic changes in response to drought stress using varieties of Tifgreen and Yukon are in progress. Comparisons of transcriptomic and proteomic profilings would provide new clues to dissect additional mechanisms responsible for the drought tolerance in bermudagrass.

Complex mechanisms such as physiological, biochemical, molecular and cellular changes might contribute to drought response of bermudagrass [6], [9], [22], [23]. Recently, Hu et al. [9] indicated that differential accumulation of dehydrins was positively connected with drought tolerance in bermudagrass. Zhao et al. [6] reported that the water-deficit tolerance of bermudagrass might be largely associated with the maintenance of photosynthesis protein metabolism and antioxidant defense. However, relationship among osmotic adaptation, ROS metabolism and antioxidant defense system has not yet been comparatively investigated in bermudagrass under drought condition. Thus, we thoroughly dissected the physiological and cellular changes among three bermudagrass varieties which showed drastically differences in drought tolerance (Yukon, SR9554 and Tifgreen). Proline is the most common compatible metabolite in the organelle and cytoplasm to regulate cell membrane stability and balance osmotic pressure of cytoplasm and environment, so higher proline content provides a significant advantage for plants to advent different stresses [6], [23]. The soluble total sugars are also important osmolytes and sucrose is the major soluble sugar in plant [6], [23]. In this study, the results indicated that proline and soluble sugar accumulations were significant higher in drought tolerant bermudagrass (Tifgreen) than those of the other varieties under water-deficit condition, leading the cells to improve the drought tolerance by osmotic adjustment (Fig. 5). Increased proline content might increase the osmotic pressure inside plant cells and cause more water uptake to keep a significantly increase in LWC. This result was consistent with previous researches which showed that proline accumulation and soluble sugars were positive related with drought tolerance in many plant species [23], [24], [25]. Moreover, water loss is a key for plant survival during drought stress condition. The results of rapid leaf water loss (Fig. 1) were consistent with changes of long term leaf water content (Fig. 4), i.e. drought tolerant bermudagrass variety exhibited lower leaf water loss, and therefore higher leaf water content. Higher amount of osmolytes accumulation in tolerant varieties might regulate plant stomatal movement to reduce water loss.

Drought stress can cause oxidative stress via rapid and excessive production of ROS, and H_2_O_2_ is the most important one [26], [27], [28]. The over-production of H_2_O_2_ can lead to oxidative damages by oxidizing proteins, damaging nucleic acids and causing lipid peroxidation (MDA), etc [26], [27], [28]. To scavenge the over-production of ROS and protect plant cells from ROS damage, plant developed complex antioxidant defense systems, including antioxidant enzymes like ascorbate peroxidase (APX), SOD, CAT, POD, GR, GPX, dehydroascorbate reductase (DHAR), monodehydroascorbate reductase (MDHAR), etc [6], [26], [27], [28]. SOD functions as the first stage of the antioxidant defense system by catalysing O_2_^−^. into H_2_O_2_ and O_2_, while APX, CAT, POD, GR and GPX are also essential for break down H_2_O_2_ through different pathway [26], [27], [28]. Using 2-DE and MS, Zhao et al. [6] found that the abundance levels of antioxidant defense proteins (APX, SOD, DHAR and MDHAR) showed a greater extent of increase in response to water-deficit stress in Tifway (tolerant variety) than in C299 (sensitive variety), indicating that antioxidant defense systems might play a critical role in the drought stress response of bermudagrass. Considering the various types of oxidative thiol modifications that may affect the antioxidant enzyme activities especially under oxidative stress conditions [29], [30], we directly measured antioxidant enzymes activities and ROS level in this study. In the drought tolerant Tifgreen plant, significant lower H_2_O_2_ and MDA levels and higher SOD, CAT, POD, GR and GPX activities were observed than those of drought sensitive Yukon variety after drought stress treatment, indicating Tifgreen showed more capacity to remove ROS levels than Yukon. Therefore, the lower ROS level (including H_2_O_2_ and MDA) and higher antioxidant enzymes activities during water-deficit period might partially contribute to the enhanced drought tolerance of Tifgreen. These investigations have extended the expression pattern of antioxidant enzymes to enzyme activity level as well as ROS level which are more directly related to drought tolerance in bermudagrass.

Additionally, we analyzed the relationship of all the above parameters. Among these parameters, the LWC and MDA content showed the highest linear correlation with EL (Figure 8). The linkage between LWC and EL indicated that the regulation of plant water status might be very important for maintaining of plant cell membrane stability. Based on correlation analysis, we found that oxidative stress triggered by drought was closely related to cell membrane stability. Therefore, the cell injury during drought stress might be largely attributed to ROS production and antioxidant defense system in bermudagrass. Osmolyte like trehalose plays a direct role in eliminating H_2_O_2_ and superoxide anions (O_2_•^−^) through protecting antioxidant enzymes (such as SOD) in wheat under heat stress [31]. We speculate that higher accumulation of osmolytes including proline and total sugars in tolerant variety (Tifgreen) (Fig. 5) might regulate ROS level through protecting of antioxidant enzymes, and the effective osmotic adjustment might alleviate drought induced cell injury. Following gradual loss of LWC under drought stress condition, more osmolyte accumulation in tolerant Tifgreen variety alleviated oxidative stress through activation of antioxidant defense system, resulting in lower lipid peroxidation, less cell injury, and higher plant survival rate.

Besides bermudagrass, there are also complex mechanisms which contribute to natural variation of drought tolerance in other species including Arabidopsis [14], [32] and *Brachypodium distachyon* [3]. Arabidopsis showed interesting phenotypic variations in response to mild water deficit [14]. The comparison of drought tolerant variety (C24) and drought sensitive varieties (Col-0, Ws-0 and Ws-2) suggested that in Arabidopsis, increasing the leaf number with a concomitant reduction of individual leaf area and increasing in leaf temperature with reduction in stomatal conductance, are associated with the enhanced drought tolerance [32]. In *B. distachyon*, the drought tolerant varieties showed little leaf wilting and fewer reduction in chlorophyll fluorescence (Fv/Fm) and LWC, while the most susceptible varieties had opposite effect in these parameters [3]. Although drought stress significantly increased the content of total soluble sugars, no significant differences were observed among different varieties of *B. distachyon* [3]. Based on these researches and our observations, different plant species might activate similar physiological and cellular responses under drought stress condition, like maintaining of LWC. However, other parameters such as total soluble sugars might contribute to different extend of variation of drought tolerance in some plant species, like bermudagrass and *B. distachyon*. As shown in this study, natural variation of drought stress tolerance in bermudagrass varieties might be largely related to the induced changes of some important parameters, especially LWC, ROS level and antioxidant defense system.

In summary, three groups of bermudagrass germplasm resources differing in drought tolerance were characterized in this study. Three varieties of Yukon, SR9554 and Tifgreen showing relatively different drought tolerance were chosen for further study to reveal the relationships between drought tolerance and changes of physiological parameters. The Tifgreen was more effective than Yukon and SR9554 in alleviating drought stress induced EL level, H_2_O_2_ content and lipid peroxidation by enhancing the accumulation of osmolytes and activities of antioxidant enzymes, and by maintaining relatively higher water status. These results suggested that changes of water status, osmolyte accumulation and antioxidant defense system during drought stress may contribute to the natural variation of bermudagrass drought tolerance. The identified bermudagrass tolerant and susceptible varieties, as well as characterized physiological parameters, can be further used in plant breeding programs to enhance drought tolerance in bermudagrass and other turfgrass species.

## Materials and Methods

### Plant materials and growth conditions

The following nine bermudagrass varieties were used in this study: Wrangler, SR9554, Yukon, LaPaloma, LaPrima (Blend of LaPaloma and SR9554), Veracruz, Riviera, Tifgreen and Tifway. Among these varieties, the seeds of Wrangler, LaPaloma, Veracruz and Riviera were kindly provided by American Johnston Seed Company (http://www.jeinc.com/seed), and those of SR9554, Yukon and LaPrima were obtained from American Seed Research of Oregon Company (http://www.sroseed.com/). Tifgreen and Tifway materials were from Wuhan Green Garden Turfgrass Company (http://www.whcp66.com/).

The seeds were stratified at 4°C for 4 days in darkness and then cultured in the sands in the growth room controlled at an irradiance of about 150 µmol quanta m^−2^ s^−1^, 25±2°C, 65%–75% relative humidity, and 16 h light and 8 h dark cycles for 60 d. During the 60 d growth, the plants were given irrigated nutrient solution two times every week. The cutting shoot-tips from the above nine bermudagrass varieties were placed in the growth room for about 1 month, and healthy cuttings with nearly the same crown size were chosen and replanted for stress treatment.

### Drought treatment and experimental design

To assess potential drought tolerance of nine varieties of bermudagrass, the healthy cuttings in pots with nearly the same crown size were subjected to watering condition (control) and drought condition (soil water deficit) by withholding water in the soil for 28 d. More than four pots of plants from each variety were used as replicates in each independent experiment, and all these pots of plants were conducted in a randomized complete block design with the same growth condition. To minimize the environment effects, the pots with soil and plants were rotated daily. The survival rate of stressed bermudagrass was recorded at 7 d after re-watering. The leaf samples were harvest at 7 d, 14 d, 21 d and 28 d after treatments for physiological parameter analyses.

### Assay of electrolyte leakage

For the EL assay, about 0.1 g leaves were incubated in 10 ml of deionized water, and shaken at room temperature for 6 h. The initial conductivity (C_i_) was measured using a conductivity meter (Leici-DDS-307A, Shanghai, China). The samples were then boiled for 20 min to completely induce all electrolytes. After cooling to room temperature, the conductivity of the killed tissues (C_max_) was determined. Relative EL (%) = (C_i_/C_max_)×100.

### Assessment of water loss and leaf water content

To access leaf water status, leaf water loss *in vitro* and LWC *in vivo* were conducted in this study. Leaf water loss was expressed as % change in leaf FW *in vitro*, and LWC was the measurement of leaf water potential *in vivo*.

To compare the water loss between nine varieties of bermudagrass, the detached leaves grown under normal conditions were placed on the weighing paper inside the same growth room. FW of the detached leaves were quantified every 1 hour intervals for up to 8 h. The water loss was then calculated from the decrease in the rate of FW at designated time intervals [25].

For LWC analysis, the leaf samples were harvest at different timepoints (7 d, 14 d, 21 d and 28 d) under control and drought treatment conditions. The FW was determined immediately after harvest and the dry weight (DW) was determined after 16 h incubation at 80°C. LWC was measured according to previously described method using the following formula: LWC (%) = (FW-DW)/FW×100 [3].

### Measurement of proline content

Proline content was estimated using described method with L-proline as standard [25]. Briefly, 0.5 g leaf samples were extracted in 3% (w/v) sulfosalicylic acid before 2 ml of ninhydrin reagent and 2 ml of glacial acetic acid were added. Well mixed solutions were boiled at 100°C for 40 min. After cooling to room temperature, the proline level of samples was calculated at 520 nm absorbance by making the specification curve with known concentration of L-proline.

### Measurement of sucrose and soluble total sugars

The sucrose and soluble total sugars were determined using the anthrone method as previous described with some modifications [23]. Briefly, 100 mg dried leaf samples were extracted in 5 ml of 80% (v/v) ethanol at 80°C for 40 min and centrifuged at 12000 rpm for 10 min. The pellets were further extracted twice with another 5 ml of 80% (v/v) ethanol. The combined supernatants were dipigmented by litter activated charcoal at 80°C for 30 min. The filtrated solution was brought to 10 ml for further analysis.

For sucrose content assay, 0.2 ml mixture (0.1 ml of the above extracts mixed with 0.1 ml of 2 M NaOH) was boiled at 100°C for 10 min. After cooling down, 1.4 ml of 30% (w/v) HCl and 0.4 ml of 0.1% (w/v) hydroxyphenol (0.1 g hydroxyphenol was dissolved in 100 ml of 80% (v/v) ethanol) were added and mixed thoroughly, and the mixture was heated at 80°C for 10 min. After cooling to room temperature, the sucrose content of sample was calculated at 480 nm of absorbance by making the specification curve with known concentration of surcose.

For the determination of soluble total sugars, the mixture of 0.1 ml of the extracts and 3 ml of 0.15% (w/v) anthrone reagent (0.3 g anthrone was dissolved in 200 ml of 7.74 M H_2_SO_4_) was heated at 90°C for 20 min. Then soluble total sugar level was examined at 620 nm of absorbance by making the specification curve with known concentration of glucose.

### Determination of lipid peroxidation

The lipid peroxidation was determined by measuring the amount of MDA, with the thiobarbituric acid (TBA) as reported previously [25]. Totally 0.5 g plant leaves were ground in 2.5 ml of regent (0.25% (w/v) TBA in 10% (w/v) trichloroacetic acid), and then boiled at 100°C for 20 min. The MDA content was determined by subtracting the non-specific absorption at 600 nm from the absorbance of the sample supernatant at 532 nm.

### Protein extraction and quantification

For plant protein extraction, about 1 g fresh leaves were ground with liquid nitrogen and then homogenized in extraction buffer (50 mM sodium phosphate buffer, pH 7.8). After centrifuging at 12000 rpm for 30 min at 4°C, the supernatant was used for protein content, H_2_O_2_ content and antioxidant enzyme activity determination [10]. The protein content was quantified using Bradford method with bovine serum albumin (BSA) as standard.

### Determination of H_2_O_2_ level

The H_2_O_2_ content was determined using method described by Hu et al. [15]. Briefly, 1 ml of the above supernatant was mixed with 1 ml of 0.1% titanium sulphate in 20% H_2_SO_4_ (v/v) thoroughly for 10 min. After centrifuged at 12000 rpm for 10 min at room temperature, the absorbance of the supernatant was measured at 410 nm using known concentration of H_2_O_2_ as control.

### Assay of antioxidant enzyme activities

The SOD (EC 1.15.1.1), CAT (EC 1.11.1.6), GR (EC 1.6.4.2), GPX (EC 1.11.1.9) activities in the samples were determined using Total SOD Assay Kit with WST-1 (S0102, Beyotime, China), CAT Assay Kit (S0051, Beyotime, China), GR Assay Kit (S0055, Beyotime, China) and Total GPX Assay Kit (S0058, Beyotime, China), respectively, according to the manufacturer's instructions. The POD (EC 1.11.1.7) activity was measured with Plant POD Assay Kit (A084-3, Nanjing Jiancheng Bioengineering Institute, China) as the instruction described.

For the determination of SOD activity, 2-(4-iodophenyl)-3-(4-nitrophenyl)-5-(2, 4-disulfophenyl)-2H-tetrazolium (WST-1) method was used [33]. WST-1 can couple with xanthine oxidase (XO) to generate O_2_^−^ and formazan dye, however, this reaction can be inhibited by SOD by catalysing O_2_^−^ into H_2_O_2_ and O_2_. Therefore, the SOD activity can be calculated by measuring the absorbance of formazan dye at 450 nm.

The CAT activity was assayed using CAT Assay Kit (S0051, Beyotime, China) as previous described [34]. Briefly, the protein supernatants were treated with excess H_2_O_2_ for decomposition by CAT for 5 min, and the remaining H_2_O_2_ coupled with a substrate was treated with POD to generate a red product, N-4-antipyryl-3-chloro-5-sulfonate-p-benzoquinonemonoimine, which can be examined at 520 nm. CAT activity can be determined by measuring the decomposition of H_2_O_2_.

For the GR activity assay, GR Assay Kit (S0055, Beyotime, China) was used. The reaction mixture included 20 µl of sample, 100 µl of GSSG solution, 70 µl of GR assay solution and 10 µl of 2 mM NADPH, and the blank was set without sample. Glutathione disulfide (GSSG) can be catalyzed to reduced glutathione (GSH) by GR in the present of NADPH. Then the GR activity can be determined by measuring the reduction of NADPH from the absorbance at 340 nm.

The GPX activity was determined using Total GPX Assay Kit (S0058, Beyotime, China) as described by Wang et al. [35]. Briefly, the reaction mixture contained 10 µl of sample supernatant, 176 µl GPX assay buffer, 10 µl GPX assay working solution (4.8 mM NADPH, 40.4 mM GSH, and GR solution supplied by the kit), 4 µl of 15 mM cumene hydroperoxide (Cum-OOH), and two controls were set without sample and without Cum-OOH, respectively. The GPX activity was calculated by measuring the reduction of NADPH to NADP^+^ at 340 nm of absorbance.

The POD activity was assayed with Plant POD Assay Kit (A084-3, Nanjing Jiancheng Bioengineering Institute, China) as the instruction described based on the guaiacol oxidation [36]. The POD activity was determined by examining the absorbance of reaction buffer at 420 nm.

The relative activities of the above antioxidant enzymes were quantified as fold change in relative to Yukon under control condition for 7 d.

### Cluster analysis

To identify the groups of bermudagrass varieties differing in sensitivities to drought stress, the data of EL, survival rate, LWC after drought treatment for 28 d, and water loss at 8 h after detachment from all nine bermudagrass varieties were chosen for cluster analysis. Hierarchical cluster analyses were performed using the CLUSTER program (http://bonsai.ims.u-tokyo.ac.jp/~mdehoon/software/cluster/) [37] by the uncentred matrix and complete linkage method. Resulting tree figures were displayed using the software package, Java Treeview (http://jtreeview.sourceforge.net/) as described by Chan et al. [38].

### Statistical analysis

All experiments in this study were repeated at least three times, and the results shown are mean ± SE of these independent experiments. For each independent experiment, at least 30 plants were used for each variety and treatment combination. Asterisk symbols above the columns in the figures indicate significant differences from Yukon at *P*<0.05 (Student's *t*-test).
